# Fatty acid synthase phosphorylation: a novel therapeutic target in HER2-overexpressing breast cancer cells

**DOI:** 10.1186/bcr2777

**Published:** 2010-11-16

**Authors:** Quanri Jin, Linda X Yuan, Delphine Boulbes, Jong Min Baek, Ying Nai Wang, Daniel Gomez-Cabello, David H Hawke, Sai Ching Yeung, Mong Hong Lee, Gabriel N Hortobagyi, Mien Chie Hung, Francisco J Esteva

**Affiliations:** 1 Department of Breast Medical Oncology, The University of Texas M. D. Anderson Cancer Center, 1515 Holcombe Blvd., Houston, TX 77030, USA; 2 Department of Molecular and Cellular Oncology, The University of Texas M. D. Anderson Cancer Center, 1515 Holcombe Blvd., Houston, TX 77030, USA; 3 Department of Molecular Pathology, The University of Texas M. D. Anderson Cancer Center, 1515 Holcombe Blvd., Houston, TX 77030, USA; 4 Department of General Internal Medicine, The University of Texas M. D. Anderson Cancer Center, 1515 Holcombe Blvd., Houston, TX 77030, USA; 5 The University of Texas Graduate School of Biomedical Sciences at Houston, 6767 Bertner Ave., Houston, TX 77030, USA; 6 Current address: Laboratory of Individualized Therapies, M. D. Anderson International España, Calle de Arturo Soria, 28033 Madrid, Spain

## Abstract

**Introduction:**

The human epidermal growth factor receptor 2 (HER2) is a validated therapeutic target in breast cancer. Heterodimerization of HER2 with other HER family members results in enhanced tyrosine phosphorylation and activation of signal transduction pathways. HER2 overexpression increases the translation of fatty acid synthase (FASN), and FASN overexpression markedly increases HER2 signaling, which results in enhanced cell growth. However, the molecular mechanism and regulation of HER2 and FASN interaction are not well defined. Lapatinib is a small-molecule tyrosine kinase inhibitor that blocks phosphorylation of the epidermal growth factor receptor and HER2 in breast cancer cells, resulting in apoptosis. We hypothesized that FASN is directly phosphorylated by HER2, resulting in enhanced signaling and tumor progression in breast cancer cells.

**Methods:**

Using mass spectrometry, we identified FASN as one of the proteins that is dephosphorylated by lapatinib in SKBR3 breast cancer cells. Immunofluorescence, immunoprecipitation, Western blotting, a kinase assay, a FASN enzymatic activity assay, an invasion assay, a cell viability assay and zymography were used to determine the role of FASN phosphorylation in invasion of SKBR3 and BT474 cells. The FASN inhibitor C75 and small interfering RNA were used to downregulate FASN expression and/or activity.

**Results:**

Our data demonstrated that FASN is phosphorylated when it is in complex with HER2. FASN phosphorylation was induced by heregulin in HER2-overexpressing SKBR3 and BT474 breast cancer cells. Heregulin-induced FASN phosphorylation resulted in increased FASN enzymatic activity, which was inhibited by lapatinib. The FASN inhibitor C75 suppressed FASN activity by directly inhibiting HER2 and FASN phosphorylation. Blocking FASN phosphorylation and activity by lapatinib or C75 suppressed the activity of matrix metallopeptidase 9 and inhibited invasion of SKBR3 and BT474 cells.

**Conclusions:**

FASN phosphorylation by HER2 plays an important role in breast cancer progression and may be a novel therapeutic target in HER2-overexpressing breast cancer cells.

## Introduction

The human epidermal growth factor receptor 2 (*HER2*) gene encodes a 185-kDa transmembrane protein that belongs to the type I family of growth factor receptors, which also includes the epidermal growth factor receptor (EGFR), HER3 and HER4. HER2 is overexpressed in 20 to 25% of invasive breast cancers, and patients with those cancers have worse overall survival and time to relapse than patients whose tumors express normal levels of HER2 [1]. This strong link between HER2 activity and the malignant process has made this protein an excellent target for studies focusing on the development of new cancer therapies [2]. The monoclonal antibody trastuzumab and the small-molecule tyrosine kinase inhibitor lapatinib are the first two HER2-targeted therapies approved by the U.S. Food and Drug Administration for treatment of patients with HER2-overexpressing breast cancer [3-5].

HER3 and HER4 bind to the well-characterized ligand heregulin (HRG), but no ligand has been identified for the extracellular domain of HER2. One of the mechanisms of HER2 activation is its heterodimerization with other HER family members. For example, upon ligand binding (that is, with HRG), HER3 can form heterodimers with HER2, resulting in markedly enhanced tyrosine phosphorylation of HER2 [6] and subsequent transphosphorylation of HER3 [7]. Like EGFR, HER2 has intrinsic tyrosine kinase activity and can interact with various cellular proteins, thereby mediating multiple signal transduction pathways, including the phosphatidylinositol-3-OH-kinase (PI3K) and mitogen-activated protein kinase pathways [8-10].

The survival of breast cancer cells, especially those with *HER2 *gene amplification, is heavily dependent on lipid metabolism [11]. HER2 overexpression increases translation of fatty acid synthase (FASN) [12,13], and FASN overexpression markedly increases EGFR and HER2 signaling, which results in enhanced cell growth [14]. Thus, FASN overexpression has been associated with poor prognosis in breast cancer patients [15]. Kumar-Sinha *et al. *originally discovered a molecular connection between FASN and HER2. These authors showed that HER2 mediates FASN induction by activating the FASN promoter via a PI3K-dependent pathway [16]. Menendez *et al. *suggested that HER2 overexpression could upregulate *FASN *gene transcription, protein synthesis, protein stability and possibly phosphorylation [17] and later showed that FASN inhibitors (for example, cerulenin and C75) and small interfering RNAs (siRNAs) targeting FASN specifically suppressed HER2 protein and mRNA expression by upregulating the HER2-specific transcriptional repressor PEA3 [18]. Pharmacologically induced or siRNA-induced inhibition of FASN in breast cancer cells results in major changes in the synthesis of phospholipids that, in turn, impair the proper localization of HER2 to the cell membrane [19]. Recent findings have shown that FASN expression is induced by HER2 overexpression, which activates the PI3K/AKT pathway and subsequently stimulates the mammalian target of rapamycin, leading to increased FASN translation [12,16,20]. HER2 can also activate sterol regulatory element binding protein-1c, resulting in the upregulation of FASN transcription [19]. Agents that target the HER2/PI3K/mammalian target of rapamycin pathway have been shown to consequently downregulate FASN expression [12,21]; conversely, FASN inhibitors block the protein expression and kinase activity of HER2 [18,22]. Blocking both FASN and HER2 results in the synergistic suppression of breast cancer cell growth [18]. These studies suggest a potential role for FASN in apoptosis, cell proliferation and invasion in HER2-overexpressing breast cancer cells and indicate the possibility that agents targeting FASN could be useful for treating breast cancers, especially those in which HER2 is overexpressed.

In this study, we further investigated the relationship between HER2-regulated FASN tyrosine phosphorylation and FASN activity, as well as the role of FASN in promoting cell invasion in HER2-overexpressing breast cancer cells. Our data support a potential role for FASN phosphorylation as a novel therapeutic target in HER2-overexpressing breast cancer.

## Materials and methods

### Cell culture and reagents

SKBR3 and BT474 breast cancer were purchased from American Type Culture Collection (Manassas, VA, USA) and grown in cell culture medium (McCoy's 5A medium (Sigma-Aldrich, St. Louis, MO, USA) for SKBR3 cells and RPMI-1640 medium (Sigma-Aldrich) for BT474 cells) supplemented with 10% fetal bovine serum, 62.5 μg/mL penicillin and 100 μg/mL streptomycin (Sigma-Aldrich) for the specified times at 37°C in a humidified atmosphere of 5% CO_2_. For the analysis of HER2 signal transduction, cells were incubated in serum-free medium overnight and then treated with the HER2 inhibitor lapatinib (ChemieTek, Indianapolis, IN, USA), the FASN inhibitor C75 (Sigma-Aldrich), epidermal growth factor (EGF; Sigma-Aldrich), insulin-like growth factor 1 (IGF-1; Sigma-Aldrich) and/or human recombinant HRG (Sigma-Aldrich) as indicated.

### Mass spectrometry

To identify the proteins that are dephosphorylated as a result of suppression of HER2, we immunoprecipitated proteins with a monoclonal antibody detecting tyrosine phosphorylation at residue 66 (PT66) and the lysates of SKBR3 breast cancer cells that had been left untreated or treated with lapatinib for 18 hours. The eluted proteins were separated by sodium dodecyl sulfate-polyacrylamide gel electrophoresis (SDS-PAGE), and the resulting gel was cut into two identical halves. One half of the gel was subjected to silver staining (Additional file 1A); the other half was subjected to Western blot analysis (Additional file 1B). Silver-stained bands from the untreated cells (control) were excised. These bands corresponded to bands in the Western blot that showed decreased expression in the cells treated with lapatinib versus untreated control. The protein bands from the silver-stained gel were subjected to analysis with an LTQ XL linear ion-trap mass spectrometer (Thermo Scientific, West Palm Beach, FL, USA) with an UltiMate 3000 high-performance liquid chromatography (Dionex, Bannockburn, IL, USA) reverse-phase column (LC-Packings C18 PepMap (Dionex); 75 μm × 150 mm) connected. Mascot software (Matrix Science, Boston, MA, USA) was used to search the mass spectra against the National Center for Biotechnology Information database.

### Kinase assay

For kinase assay, the FASN substrate was prepared by immunoprecipitation. SKBR3 cells were incubated in serum-free medium overnight, treated with 1 μM lapatinib for one hour to minimize the effect of HER2 kinase and lysed with a lysis buffer. We used an anti-FASN antibody (Santa Cruz Biotechnology, Santa Cruz, CA, USA) and protein G agarose beads (Millipore, Billerica, MA) to immunoprecipitate FASN. Endogenous HER2 kinase was immunoprecipitated from the lysates of SKBR3 cells treated with: 1) no agent (untreated control); 2) 50 ng/mL HRG for 15 minutes; 3) 1 μM lapatinib for one hour, or 4) 1 μM lapatinib for one hour followed by 50 ng/mL HRG for 15 minutes. To measure kinase activity, we mixed the FASN substrate and HER2 kinase (endogenous HER2 or human recombinant HER2 (BPS Bioscience Inc., San Diego, CA, USA) as positive control) in kinase reaction buffer (10 mM MnCl_2_, 5 μC ^32^P-γ- adenosine-5'-triphosphate (ATP), 20 mM Tris-Cl [pH, 7.5], 50 mM NaCl, 10 mM MgCl_2_, 1 mM NaF, 1 mM Na_3_VO_4_, 20 mM β-glycerophosphate, 1 mM dithiothreitol and 20 μM ATP) for 30 minutes at 30°C. Phosphorylated FASN was assessed by autoradiography following SDS-PAGE.

### Immunoprecipitation and Western blot analysis

SKBR3 cells were treated for one hour with: 1) medium only (control); 2) 50 ng/mL HRG; 3) 1 μM lapatinib; or 4) 50 ng/mL HRG plus 1 μM lapatinib. BT474 cells were treated with: 1) medium only (control); 2) 50 ng/mL HRG for one hour; 3) 10 μM C75 for six hours; or 4) 50 ng/mL HRG for one hour plus 10 μM C75 pretreatment for five hours. Cells were lysed in lysis buffer containing 1% Triton-X 100, 50 mM Tris-Cl (pH, 8.0), 150 mM NaCl, 5 mM ethylenediaminetetraacetic acid (pH, 8.0), 50 mM NaF, 1 mM Na3VO4, 50 mM β-glycerophosphate, a protease inhibitor cocktail (Roche, Indianapolis, IN, USA) and a phosphatase inhibitor cocktail (Calbiochem-EMD Biosciences, San Diego, CA, USA). FASN proteins were immunoprecipitated via 2 μg anti-FASN antibody and a 50% protein G agarose bead slurry, and the immunoprecipitated samples were boiled and analyzed by Western blotting as previously described [23]. Whole-cell lysates were subjected to Western blotting as previously described [24]. The following antibodies were used for Western blot analysis: mouse monoclonal antibodies detecting tyrosine phosphorylation (PT66) and β-actin (Sigma-Aldrich); rabbit polyclonal antibodies detecting pHER2 (Tyr 1248), pHER3 (Tyr 1289), pAkt (S473), pErk1/2, Akt, Erk1/2 and HER3 (Cell Signaling Technology, Inc., Danvers, MA, USA); mouse monoclonal antibodies detecting FASN, HER2 and HER3 (Santa Cruz) and an Alexa Flour 680 goat anti-mouse immunoglobulin (IgG) antibody, Alexa Flour 680 goat anti-rabbit IgG antibody and Alexa Flour 680 donkey anti-goat IgG antibody (Invitrogen Corporation, Carlsbad, CA, USA). The membranes were washed extensively, and the proteins were visualized and quantified using Odyssey imaging software v1.2 (LI-COR Inc., Lincoln, NE, USA). Representative results from three separate experiments are reported.

### Immunofluorescence analysis of subcellular protein distribution

Modified immunofluorescence analysis was performed as described previously [25]. Briefly, SKBR3 cells were trypsinized and seeded onto a Nunc six-well Lab-Tek chamber slide (Thermo Scientific) at a density of 1 × 10^5 ^cells/well and cultured overnight. After 12 hours of serum starvation, the culture medium was changed to serum-free medium containing either untreated control or 50 ng/mL HRG and the cells were treated for an additional three hours. After treatment, cells were fixed with 2% paraformaldehyde, permeabilized with ice-cold methanol and blocked with 3% bovine serum albumin solution in triethanolamine buffered saline containing 0.1% Tween 20 (TBST). Chamber slides were incubated with mouse anti-FASN and rabbit anti-HER2 primary antibodies (1:100 in phosphate-buffered saline-Tween 20, Santa Cruz) overnight at 4°C. Slides were rinsed three times with TBST and incubated with Alexa 488 (Ex 495/Em 519) donkey anti-mouse and Alexa 594 (Ex 590/Em 617) donkey anti-rabbit secondary antibodies (Invitrogen) for 30 minutes at room temperature. Finally, slides were washed three times in TBST, rinsed with distilled water and mounted with Faramount mounting medium (Dako North America Inc., Carpinteria, CA, USA). Slides were viewed and photographed using a Zeiss LSM 710 immunofluorescence microscope.

### siRNA transfection

Transient transfection of siRNAs was performed with Oligofectamine reagent (Invitrogen) according to the manufacturer's instructions. Briefly, SKBR3 and BT474 cells were plated in six-well plates at a density of 3 × 10^5 ^cells/well. Either 10 μL of 20 μM FASN siRNA (si-FASN, Thermo Scientific) or 10 μL of scrambled control siRNA (Dharmacon Research Inc.) was transfected to each well, and cells were incubated for 24 hours at 37°C in a humidified CO_2 _incubator. Transfected cells were used for Western blotting or cell invasion analyses.

### FASN enzymatic activity assay

A modified FASN enzymatic activity assay was performed as previously described [20]. Briefly, after overnight serum starvation, SKBR3 and BT474 cells were incubated for 12 hours in serum-free medium containing 1 μCi/mL (2-^14^C)acetate and no agent (control), 50 ng/mL HRG, 1 μM lapatinib, 10 μM C75, 50 ng/mL HRG plus 10 μM C75 or 50 ng/mL HRG plus 1 μM lapatinib. Cells were harvested in 1 × phosphate-buffered saline. The (2-^14^C) incorporated lipid was extracted with a solution of chloroform and methanol (1:3) after treatment with ddH_2_O and methanol (6:5). After being shaken for 30 minutes at room temperature, samples were centrifuged at 12,500 × g for five minutes, and the lower phase was counted for fatty acid incorporation. Changes in FASN activity were reported as a percentage compared to untreated control cells. Each experiment was repeated in triplicate.

### Tumor cell invasion assay

To detect cell invasion, we used a QCM ndothelial cell matrix cell invasion assay with a 24-well (8-μm pores) fluorometric matrigel invasion chamber (Millipore). To investigate the role of lapatinib in cell invasion, we treated SKBR3 and BT474 cells with 1) medium only (untreated control); 2) 0.2 μM lapatinib; 3) 50 ng/mL HRG; or 4) 50 ng/mL HRG plus 0.2 μM lapatinib. To investigate the role of C75 in cell invasion, we treated both SKBR3 and BT474 cells with: 1) medium only (untreated control); 2) 10 μM C75; 3) 50 ng/mL HRG; or 4) 50 ng/mL HRG plus 10 μM C75. To evaluate the role of si-FASN in cell invasion, we transfected both SKBR3 and BT474 cells with scrambled siRNA or si-FASN for 24 hours. After 12 hours of serum starvation, the transfected SKBR3 and BT474 cells were trypsinized and seeded at a density of 1 × 10^5 ^cells/well and then incubated in serum-free medium with or without 50 ng/mL HRG. In the bottom well, 10% fetal bovine serum medium was used as a chemoattractant. Each experiment was performed in triplicate. After 36 hours of incubation, the cells remaining above the insert membrane were removed by gentle scraping with a sterile cotton swab. Cells that invaded through the matrigel to the bottom of the insert were fixed and stained with crystal violet. Invading cells on representative sections of each membrane were counted under light microscopy. Under each treatment condition, the number of invading cells was calculated as the percentage of invaded cells compared with the control cultures. All experiments were done at least thrice.

### Zymography

SKBR3 and BT474 cells were incubated in serum-free medium for an additional 12 hours after the cell invasion assay and treated with no agent (control), 50 ng/mL HRG, 1 μM lapatinib, 10 μM C75, 50 ng/mL HRG plus 10 μM C75 or 50 ng/mL HRG plus 1 μM lapatinib. Matrix metallopeptidase 9 (MMP-9) has been associated with invasion in HER2-overexpressing breast cancer cells. To evaluate the effect of si-FASN on MMP-9 activity, we seeded SKBR3 and BT474 cells in 100-mm dishes at a density of 1 × 10^6 ^cells/well; the next day, the cells were transfected with either scrambled siRNA or si-FASN for 24 hours. Then the transfected SKBR3 cells were incubated in serum-free medium for an additional 12 hours and treated with: 1) no agent (control) or 2) 50 ng/mL HRG. After 24 hours of treatment, supernatant medium was collected, concentrated (from 8 mL to 200 μL) by the use of an Amicon Ultra-15 centrifugal filter (Millipore) and stored at -80°C for use in a zymogram assay. Cells were harvested for Western blot analysis. A modified zymogram assay was performed as previously described [26]. The protease activity of MMP-9 was assessed by SDS-PAGE with 8% zymogram gels containing 0.2% (2 mg/mL) gelatin. Areas of protease activity appeared as clear bands against a dark Coomassie blue background where the protease had digested the substrate.

### Cell viability assay

SKBR3 and BT474 cells were trypsinized, counted and seeded at a density of 8 × 10^3 ^cells/well on a 96-well plate overnight. The next day, cells were treated with 0.2 μM lapatinib or 10 μM C75 with, or without, 50 ng/mL HRG for an additional 36 hours. Cell viability was assessed with a CellTiter-Glo luminescent cell viability assay kit (Promega, Madison, WI, USA) according to the manufacturer's instructions.

### Statistical analysis

Data from FASN activity, cell viability and cell invasion assays were analyzed using the Student's two-tailed *t-*test. *P *< 0.05 was considered statistically significant.

## Results

### FASN is phosphorylated by HER2 activation in HER2-overexpressing breast cancer cells

As expected, lapatinib inhibited EGFR and HER2 phosphorylation in SKBR3 breast cancer cells. Lapatinib also dephosphorylated other proteins, such as FASN, Hsp90 and Hsp70. Because of the known connection between FASN and HER2 in breast cancer cells [12,16,18,27], we focused on FASN for the subsequent experiments. We further confirmed dephosphorylation of FASN by lapatinib by measuring total and phosphorylated FASN levels in HER2-positive SKBR3 and BT474 breast cancer cells with or without treatment with a clinically relevant concentration of lapatinib [28] overnight (Additional file 1C).

**Figure 1:**
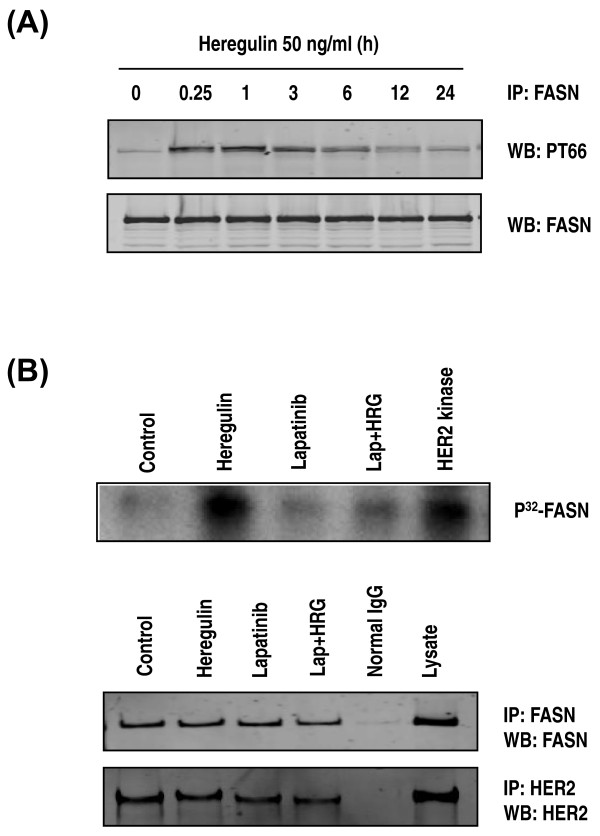
**FASN phosphorylation by HRG in SKBR3 cells**. **(A) **SKBR3 cells were incubated in serum-free medium overnight and then treated with 50 ng/mL heregulin (HRG) for the indicated times. FASN was immunoprecipitated and immunoblotted by the use of a monoclonal phosphotyrosine (PT66) and anti-FASN antibodies. **(B) **Immunoprecipitated FASN substrate was incubated with HER2 kinase and radiolabeled with (γ-P^32^)-ATP for 30 minutes at 30ºC, separated by sodium dodecyl sulfate polyacrylamide gel electrophoresis and visualized by autoradiography. Recombinant HER2 kinase was used as a positive control. Western blotting for FASN and HER2 was performed to confirm that equal amounts of FASN or HER2 were used in each kinase assay.

To determine whether FASN phosphorylation is ligand dependent, we incubated SKBR3 cells in serum-free medium overnight and then treated the cells with EGF, HRG or IGF-1 for the indicated time course. We found an increase in FASN phosphorylation upon treatment with HRG (Figure 1A) but not with EGF or IGF-1 (data not shown). This finding suggests that HER3 or HER4 is involved in FASN activation, presumably by heterodimerization with HER2 [29]. HRG-induced FASN phosphorylation and lapatinib-induced FASN dephosphorylation were confirmed by the use of an *in vitro *kinase assay with a FASN substrate (Figure 1B).

**Figure 2:**
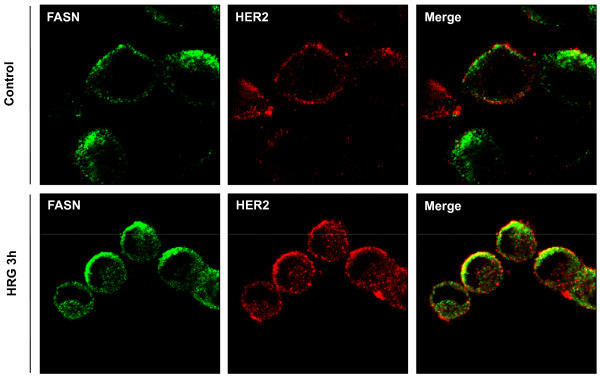
**Colocalization of FASN and HER2 in breast cancer cells**. Treatment of SKBR3 cells with HRG caused HER2 (red) and FASN (green) to colocalize at the cell membrane (yellow on merged images).

**Figure 3:**
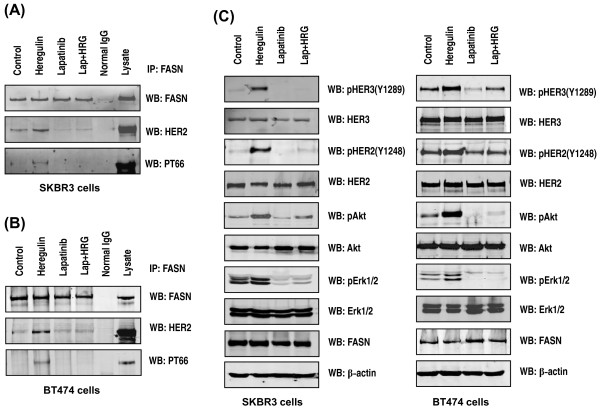
**Association of FASN and HER2 in breast cancer cells**. SKBR3 **(A) **and BT474 **(B) **cells were treated for one hour with: 1) no agent (control); 2) 50 ng/mL HRG; 3) 1 μM lapatinib or 4) 50 ng/mL HRG plus 1 μM lapatinib. The immunoprecipitated FASN complexes were subjected to Western blotting for FASN, HER2 and PT66. **(C) **Whole-cell lysates from SKBR3 (left panel) and BT474 cells (right panel) were subjected to Western blotting for pHER2 (Y1248), pHER3 (Y1289), pAkt (S473), pErk1/2, HER2, HER3, Akt, Erk1/2, FASN and β-actin.

We evaluated the HRG-induced interaction between FASN and HER2 *in vivo *with immunofluorescence microscopy. Figure 2 demonstrates that FASN and HER2 colocalized to the cell membrane upon HRG treatment. Coimmunoprecipitation of FASN and HER2 showed that HRG induced interaction between FASN and HER2 and tyrosine phosphorylation of FASN in both SKBR3 and BT474 cells (Figure 3). Concomitant with the lapatinib-induced FASN dephosphorylation the HRG-induced interaction between FASN and HER2 was also markedly suppressed by lapatinib (Figure 3A). The changes in HER2 activity induced by HRG and lapatinib were confirmed by the following findings: HRG induced the tyrosine phosphorylation of HER2, HER3, Akt and Erk1/2, which was inhibited by lapatinib, whereas neither of these agents changed the total expression of HER2, HER3, Akt, Erk1/2 and FASN in either SKBR3 (Figure 3C, left panel) or BT474 cells (Figure 3C, right panel).

### Tyrosine phosphorylation of FASN is critical for maintaining FASN activity in breast cancer cells

**Figure 4:**
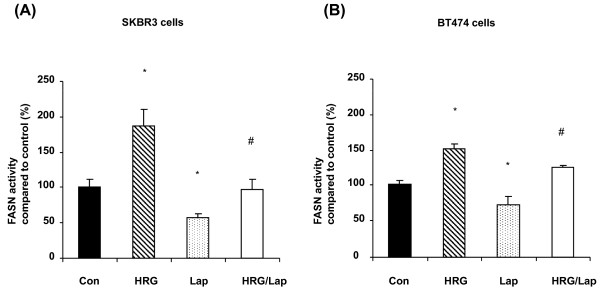
**FASN enzymatic activity of cells treated with or without lapatinib**. FASN activity was measured in SKBR3 **(A) **and BT474 **(B) **cells treated with 50 ng/mL HRG, 1 μM lapatinib or 50 ng/mL HRG plus 1 μM lapatinib. Cells were harvested, and lipids were extracted as described in Materials and methods. A simple *t*-test was used to assess the differences in FASN activity between any two experimental conditions. *, statistically significant compared with FASN activity in control cells; ^#^, statistically significant compared with FASN activity in cells treated with HRG; *P *< 0.05 was considered statistically significant.

The posttranslational modification of proteins, especially phosphorylation, is closely associated with protein biological activity [10]. We evaluated whether FASN activity could be modulated by changes in FASN tyrosine phosphorylation levels *in vivo*. SKBR3 cells treated with HRG (50 ng/mL) showed significantly increased FASN activity (*P *< 0.001), and FASN activity was markedly inhibited by lapatinib (*P *< 0.001) in both SKBR3 (Figure 4A) and BT474 cells (Figure 4B). This result shows that lapatinib suppressed both basal FASN activity and HRG-induced FASN activity (Figures 4A, B) and indicates that tyrosine phosphorylation of FASN plays a critical role in maintaining the physiological function of FASN in breast cancer cells.

### FASN inhibitor C75 suppresses FASN activity and FASN tyrosine phosphorylation in breast cancer cells

**Figure 5:**
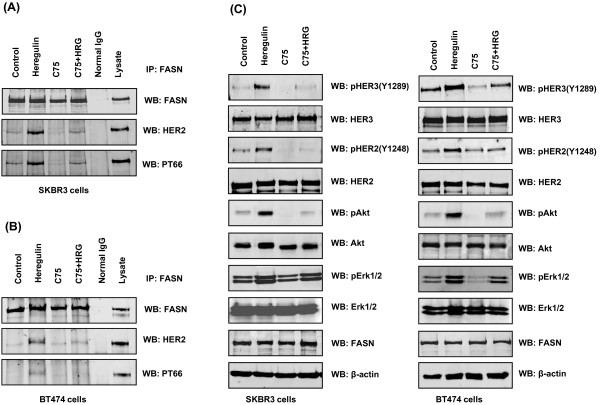
**FASN phosphorylation in breast cancer cells treated with C75**. SKBR3 **(A) **and BT474 **(B) **cells were pretreated with 10 μM C75 for five hours and then treated for one additional hour with 1) no agent (control); 2) 50 ng/mL HRG; 3) 10 μM C75; or 4) 50 ng/mL HRG plus 10 μM C75. The immunoprecipitated FASN complexes were assessed for FASN, HER2 and PT66 by Western blotting. **(C) **Whole-cell lysates from SKBR3 and BT474 cells were subjected to Western blotting for pHER2 (Y1248), pHER3 (Y1289), pAkt (S473), pErk1/2, HER2, HER3, Akt, Erk1/2, FASN and β-actin.

Since HER2 phosphorylation and activity were suppressed by FASN inhibitors in previous studies [27,30,31], we further investigated whether tyrosine phosphorylation of FASN could be inhibited by a FASN inhibitor. We found that the FASN inhibitor C75 suppressed both HRG-induced interaction between FASN and HER2 and tyrosine phosphorylation of FASN in SKBR3 (Figure 5A) and BT474 cells (Figure 5B). The changes in HER2 activity induced by HRG and C75 were confirmed by the following findings: HRG induced tyrosine phosphorylation of HER2, HER3, Akt and Erk1/2, which was inhibited by C75, whereas neither of these agents changed the total expression of HER2, HER3, Akt, Erk1/2 and FASN in both SKBR3 (Figure 5C, left panel) and BT474 cells (Figure 5C, right panel).

**Figure 6:**
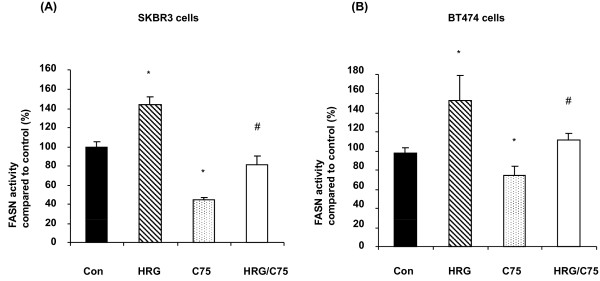
**FASN activity in breast cancer cells treated with C75**. FASN activity was measured in SKBR3 **(A) **and BT474 **(B) **cells treated with: 1) no agent (control); 2) 50 ng/mL HRG; 3) 10 μM C75 or 4) 50 ng/mL HRG plus 10 μM C75. A simple *t*-test was used to assess the differences in FASN activity between any two experimental conditions. *, statistically significant compared with FASN activity in control cells; ^#^, statistically significant compared with FASN activity in cells treated with HRG; *P *< 0.05 was considered statistically significant.

We confirmed that HRG increased FASN activity (*P *< 0.001 in SKBR3 cells and *P *< 0.01 in BT474 cells) and that FASN activity was inhibited by C75 in both SKBR3 (Figure 6A) and BT474 cells (Figure 6B). These results show that C75 suppressed both basal FASN activity (*P *< 0.01 in both cell lines) and HRG-induced FASN activity (*P *< 0.001 in SKBR3 cells and *P *< 0.01 in BT474 cells). These data indicate that C75-induced FASN dephosphorylation was involved in the suppression of FASN physiological function in breast cancer cells.

### Targeting FASN inhibits invasion of breast cancer cells

**Figure 7:**
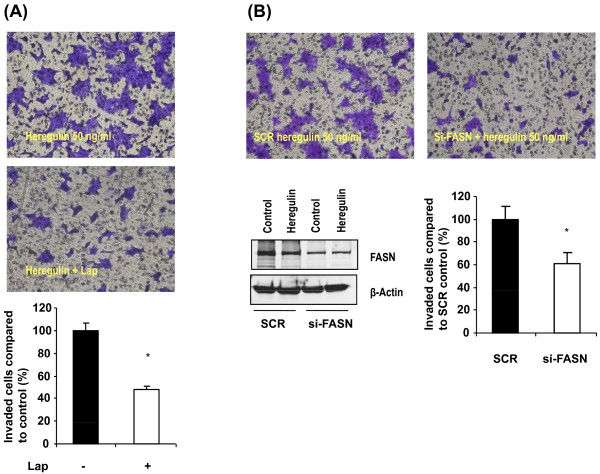
**HRG-induced invasion in HER2-positive BT474 breast cancer cells treated with lapatinib or siRNA targeting FASN**. **(A) **BT474 cells were treated with 50 ng/mL HRG or 50 ng/mL HRG plus 0.2 μM lapatinib for 36 hours. The invaded BT474 cells were stained with crystal violet and counted under a microscope. Representative microscopic images are shown. The changes in cell invasion compared with HRG-induced cell invasion are shown in a bar graph. All experiments were done three times. A simple *t*-test was used to assess differences in the number of invaded cells between any two experimental conditions. *, statistically significant compared with cells treated with HRG; *P *< 0.05 was considered statistically significant. **(B) **BT474 cells transfected with scrambled siRNA or siRNA targeting FASN (si-FASN) were treated with 50 ng/mL HRG for 36 hours. The efficiency of si-FASN knockdown was confirmed by Western blotting for FASN and β-actin (lower left). The changes in cell invasion compared with the invaded scrambled siRNA-transfected untreated cells are shown as a percentage in the bar graph (lower right). All experiments were done three times. A simple *t*-test was used to assess differences in the number of invaded cells between any two experimental conditions. *, statistically significant compared with scrambled si-RNA-transfected cells treated with HRG; *P *< 0.05 was considered statistically significant.

**Figure 8:**
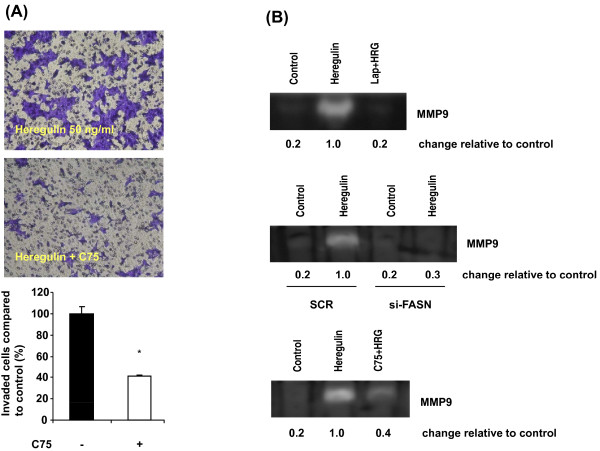
**Cell invasion and activity of MMP9 in HER2-positive BT474 breast cancer cells with suppressed FASN**. **(A) **BT474 cells were treated with 50 ng/mL HRG or 50 ng/mL HRG plus 10 μM C75 for 36 hours. Cells shown in the microscopic image represent invasive cells. The changes in cell invasion compared with HRG-induced cell invasion are shown as a percentage in the bar graph. All experiments were done three times. A simple *t*-test was used to assess differences in the number of invaded cells between any two experimental conditions. *, statistically significant compared with cells treated with HRG; *P *< 0.05 was considered statistically significant. **(B) **BT474 cells were treated as described in Figures 7A, B and 8A, and the medium was harvested for zymogram assay as described in Materials and methods. MMP-9 activity is shown as the digested clear band in Coomassie blue-stained gel containing 0.2% gelatin.

Previous studies have shown that inhibition of FASN suppresses cell survival and proliferation *in vitro *and *in vivo *[30-33]. Since invasion is one of the key steps in the metastatic process, we further investigated the role of FASN in the invasion of breast cancer cells. We evaluated the effects of lapatinib, si-FASN and C75 on the invasion of HER2-positive breast cancer cells. Lapatinib at 0.2 μM or C75 at 10 μM, with or without HRG, for 36 hours had no significant effect on cell viability (Additional file 2). Without HRG, SKBR3 and BT474 cells were minimally invasive (Additional file 3). HRG-induced cell invasion was markedly inhibited by 0.2 μM lapatinib in both SKBR3 (Additional file 3A) and BT474 cells (Figure 7A; *P *< 0.001 in both cell lines). In both SKBR3 and BT474 cells, knockdown of FASN by the transfection of si-FASN markedly inhibited HRG-induced cell invasion (*P *< 0.001) compared to invasion in scrambled siRNA-transfected cells treated with HRG (Additional file 3B and Figure 7B). Knockdown of FASN by the transfection of si-FASN was confirmed by Western blotting (Figure 7B). In both SKBR3 and BT474 cells, 10 μM C75 also significantly suppressed HRG-induced cell invasion (*P *< 0.001 in both cell lines) (Figure 8A and Additional files 3C, 4, 5). Since the activation and secretion of MMP-9, a key enzyme in the degradation of extracellular matrices that promotes tumor cell invasion, are significantly induced by HRG in SKBR3 cells [34], we performed a zymogram assay to investigate whether MMP-9 is involved in the regulation of cell invasion. We found that HRG increased the secretion and activity of MMP-9 in BT474 cells, whereas lapatinib remarkably inhibited HRG-enhanced MMP-9 activity by 80% in BT474 cells (Figure 8B). Transfection of si-FASN significantly inhibited HRG-enhanced MMP-9 activity by 70% in BT474 cells (Figure 8B). C75 markedly suppressed HRG-enhanced MMP-9 activity by 60% in BT474 cells (Figure 8B). These data indicate that targeting FASN inhibits HER2-overexpressing breast cancer cell invasion at least in part through downregulation of MMP-9 activity.

**Figure 9:**
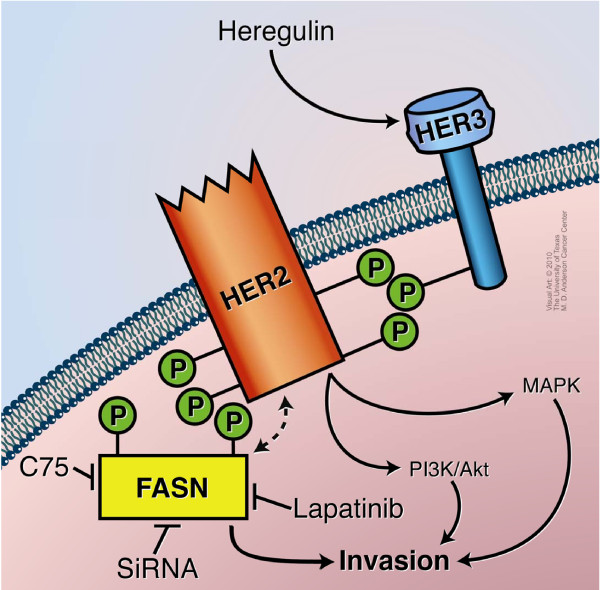
**Regulation of cell invasion through functional interaction between FASN and HER2 in HER2-overexpressing breast cancer cells**. When HRG binds to HER3, HER2 interacts with HER3 and induces tyrosine phosphorylation of HER3. The activated HER2 not only stimulates the PI3K/Akt and MAPK signaling pathways but also induces tyrosine phosphorylation of FASN and upregulation of FASN activity, leading to increased cell invasion. Suppression of FASN indirectly (for example, with lapatinib) or directly (for example, with C75 or si-FASN) inhibits invasion of HER2-overexpressing breast cancer cells.

Our findings support the following model: cell invasion was regulated by the functional interaction between FASN and HER2 in HER2-overexpressing SKBR3 and BT474 breast cancer cells (Figure 9). HRG is known to induce the interaction between HER3 and HER2, which phosphorylates HER3 [7]. In our study, the activated HER2 not only activated the PI3K/Akt and mitogen-activated protein kinase signaling pathways but also enhanced FASN activity by direct interaction and phosphorylation, leading to an increase in cell invasion. When FASN activity was suppressed by either C75 and si-FASN or lapatinib through the downregulation of HER2-induced FASN phosphorylation, cell invasion was significantly inhibited in HER2-overexpressing SKBR3 and BT474 breast cancer cells. The model proposed in Figure 9 is supported by our findings and those of others.

## Discussion

Our results support the hypothesis that HER2-mediated FASN phosphorylation plays an important role in breast cancer progression and may be a novel therapeutic target in HER2-overexpressing breast cancer cells. Lapatinib inhibits EGFR and HER2 phosphorylation in breast cancer cells [35], and it is an effective treatment for patients with HER2-positive metastatic breast cancer that has progressed after prior therapies [3]. We previously showed that lapatinib induces apoptosis in trastuzumab-resistant breast cancer cells [36]. Our current results suggest that FASN dephosphorylation plays an important role in the antitumor activity of lapatinib in HER2-positive breast cancer cells. Recently, lapatinib was shown to suppress HER2 phosphorylation and ubiquitination, resulting in a significant accumulation of inactive HER2 receptors at the cell surface, which enhanced the effect of trastuzumab-induced, antibody-dependent cellular cytotoxicity in HER2-overexpressing breast cancer cells [37]. Vazquez-Martin *et al. *[38] demonstrated that lapatinib enhanced HER2 extracelluar domain shedding and inhibited HER2 phosphorylation and activity, thereby suppressing HER2-overexpressing cancer cell growth. Our results show that lapatinib inhibits HER2-induced FASN phosphorylation and suppresses FASN activity in HER2-overexpressing breast cancer cells. Treatment with C75 or lapatinib inhibits the survival of HER2-positive breast cancer cells [30-33,36].

In many types of cancer, including breast cancer, FASN overexpression robustly induces *de novo *lipogenesis. The generated lipids are integrated into membrane lipid rafts and modulate membrane receptor tyrosine kinases (for example, the EGFR family) which, in turn, results in the initiation of oncogenic signaling pathways involving cell survival, proliferation, migration and invasion [19,39]. FASN overexpression increases EGFR and HER2 protein expression and tyrosine phosphorylation, and thereby amplifies oncogenic signaling pathways that contribute to tumorigenic transformation [14].

C75 is a competitive, irreversible inhibitor of FASN [15]. Experimental evidence has demonstrated that C75 can induce apoptosis in breast cancer cell lines and delay tumor growth in human breast cancer xenografts. The antitumor activity of C75 is not well defined at the molecular level, and it may vary depending on the model systems used. In their original study, Menendez *et al. *[18] described the mechanism of HER2 downregulation by C75. Briefly, C75 suppressed HER2 protein and mRNA expression by upregulating the HER2-specific transcriptional repressor PEA3. C75 treatment resulted in decreased phospholipid synthesis which impaired the proper localization of HER2 to the cell membrane in breast cancer cells [19]. The suppression of HER2 kinase activity by C75 results in the synergistic promotion of apoptosis induced by other HER2-directed therapies in breast cancer cells [14,22,27].

Whether FASN is regulated post-transcriptionally in cancer cells remains unclear. Several studies have reported associations between the AMP-activated protein kinase (AMPK) and fatty acid metabolism. In one study, nicotine activated AMPK in 3T3L1 adipocytes, resulting in enhanced threonine phosphorylation and consequent inhibition of FASN [40]. It is possible that different FASN phosphorylation statuses could modulate FASN activity differently; that is, threonine phosphorylation may inhibit FASN activity, whereas, tyrosine phosphorylation may increase FASN activity. Furthermore, AMPK activation could be the key factor in the cardiac-protective action of lapatinib in contrast to the cardiac toxicity of trastuzumab, which was suggested in two independent studies that demonstrated the ability of reversible dual EGFR/HER2 inhibitors (lapatinib and a lapatinib-like agent, GW2974) to activate AMPK in HER2-expressing human cardiomyocytes [41,42]. However, we did not observe lapatinib-induced AMPK activation in our experiment setting, probably because of the differences in dose (25 or 50 μM GW2974 and lapatinib in the previous studies versus 0.2 or 1.0 μM lapatinib in our experiment) or because of tissue-specific response (cardiomyocyte versus breast cancer cell). Moreover, C75 was also reported by Landree *et al. *to modulate AMPK phosphorylation and activity in neurons [43]. However, under our treatment conditions, AMPK activation was not altered, a finding that was confirmed by the absence of changes in AMPK substrate acetyl-CoA carboxylase phosphorylation (data not shown). Therefore, the AMPK-induced threonine phosphorylation of FASN likely did not play a role in the regulation of FASN activity by the relatively low dose of lapatinib or C75 we used in HER2-positive breast cancer cells.

Our data demonstrate that HRG can activate the HER2 tyrosine kinase (not the serine or threonine kinase), thus leading to interaction between HER2 and FASN and subsequently inducing the tyrosine phosphorylation of FASN in human breast cancer cells. Lapatinib and C75 abrogated the colocalization of FASN and HER2 and suppressed the tyrosine phosphorylation of FASN in both SKBR3 and BT474 cells. We showed a direct correlation between the tyrosine phosphorylation and activity of FASN in breast cancer cells. HRG enhanced FASN activity (that is, lipid synthesis), whereas lapatinib and C75 suppressed it. Since these treatments (HRG, lapatinib and C75 changed the levels of FASN protein expression in either SKBR3 or BT474 cells, the effects of these agents on FASN activity are likely directly related to changes in FASN tyrosine phosphorylation levels. Although C75 directly binds to FASN and inhibits its activity [13], in our study, C75 also suppressed HRG-induced FASN tyrosine phosphorylation, a mechanism that might be partly involved in further decreasing FASN activity. This novel finding indicates that the regulation of FASN activity by tyrosine phosphorylation may play an important role in the cell growth, invasion and metastasis of HER2-overexpressing breast cancer.

We showed that suppression of FASN by C75 or si-FASN inhibited the HRG-enhanced invasion of HER2-overexpressing breast cancer cells. HRG has been shown to induce the interaction of HER2 with HER3 and HER4, resulting in increased tyrosine phosphorylation of HER2 in breast cancer cells and, thus, increased metastasis by enhancing tumor cell invasion and intravasation *in vivo *[44]. The role of HER3 and HER4 in the regulation of FASN is unknown. However, our observation that FASN phosphorylation was induced by HRG suggests that HER3 is involved in FASN activation, presumably by heterodimerization with HER2 [45,46]. HER4 likely does not play a role in HRG-induced FASN phosphorylation, because HER4 was not detectable in BT474 and SKBR3 cells. HRG-induced activation of the HER2 signaling pathway plays an important role in the activation of MMP-9. Targeting endogenous HRG by anti-sense cDNA significantly reduces HER2 activity, inhibits MMP-9 activity and subsequently suppresses cell invasion [47]. Yao *et al. *[48] demonstrated that HRG increases MMP-9 activity, which is completely inhibited by the PKC inhibitor RO318220 and p38 kinase inhibitor SB293580 and partially suppressed by the MEK-1 inhibitor PD098059 but not blocked by the PI3K inhibitor wortmannin. In contrast, these authors found that the PI3K inhibitor LY294002 markedly blocks EGF-enhanced MMP-9 activity. In our study, we showed that inhibition of the HER2 signaling pathway by lapatinib significantly suppressed FASN activity and markedly downregulated HRG-enhanced MMP-9 activity, resulting in the inhibition of cell invasion.

## Conclusions

In summary, our data demonstrate that the inhibition of HER2 kinase activity by lapatinib results in decreased FASN tyrosine phosphorylation and reduced FASN enzymatic activity, which further downregulates HER2 signaling and results in suppressed breast cancer cell invasion. Future studies will focus on identification of the FASN tyrosine phosphorylation sites induced by HER2 and the role of HER3 in the regulation of FASN phosphorylation.

## Abbreviations

AMPK: AMP-activated protein kinase; EGFR: epidermal growth factor receptor; FASN: fatty acid synthase; HER2: human epidermal growth factor receptor 2; HER3: human epidermal growth factor receptor 3; HER4: human epidermal growth factor receptor 4; HRG: human recombinant heregulin; IgG: immunoglobulin; IGF-1: insulin-like growth factor 1; MMP-9: matrix metallopeptidase 9; PI3K: phosphatidylinositol-3-OH-kinase; PT66: tyrosine phosphorylation; siRNA: small interfering RNA; SDS-PAGE: sodium dodecyl sulfate-polyacrylamide gel electrophoresis; si-FASN: siRNA-targeting FASN.

## Competing interests

The authors declare that they have no competing interests.

## Authors' contributions

QJ and LXHY carried out molecular and cell biology studies and drafted the manuscript. DRB and YNW participated in the immunofluorescence assays. JMB and DGC participated in the cell viability and FASN enzymatic activity assays. DHH carried out the mass spectrometry analysis. SCJY, MHL, GNH and MCH participated in the design of the study and helped draft the manuscript. FJE conceived of the study, participated in its design and coordination and helped draft the manuscript. All authors read and approved the final manuscript.

## Supplementary Material

Additional file 1 **Protein tyrosine phosphorylation in HER2-positive breast cancer cells treated with lapatinib**. **(A **and **B) **SKBR3 breast cancer cells were treated with 1 μM lapatinib or DMSO (control) overnight (18 hours). Equal amounts of protein from total whole-cell lysates were immunoprecipitated with the antiphosphotyrosine antibody PT66 and separated by sodium dodecyl sulfate polyacrylamide gel electrophoresis. Steady-state protein levels of phosphotyrosine were assessed by silver staining (A) and Western blotting (B). **(C) **Phosphotyrosine immunoprecipitated complexes were obtained from SKBR3 and BT474 cells and detected by Western blotting on FASN. Click here for file

Additional file 2 **Viability of cells treated with low doses of lapabinib or C75**. SKBR3 and BT474 cells were seeded at a density of 8 × 10^3 ^cells/well on a 96-well plate. The next day, cells were treated with 0.2 μM lapatinib or 10 μM C75 with or without 50 ng/mL HRG for an additional 36 hours. Cell viability assay was performed by the use of the CellTiter-Glo luminescent cell viability assay kit (Promega Corporation) according to the manufacturer's instructions. Click here for file

Additional file 3 **Implication of FASN and HER2 in the invasion of HER2-positive SKBR3 breast cancer cells**. We assessed cell invasion using a matrigel invasion chamber as described in Materials and methods. **(A) **SKBR3 cells were treated with 50 ng/mL HRG or 50 ng/mL HRG plus 0.2 μM lapatinib for 36 hours. The changes in cell invasion compared with HRG-induced cell invasion were shown as a percentage in a bar graph. All experiments were done three times. A simple *t*-test was used to assess differences in the number of invaded cells between any two experimental conditions. *, statistically significant compared with cells treated with HRG; *P *< 0.05 was considered statistically significant. **(B) **SKBR3 cells transfected with either scrambled siRNA or si-FASN were treated with 50 ng/mL HRG for 36 hours. The efficiency of si-FASN knockdown was confirmed by Western blotting for FASN and β-actin. The changes in cell invasion compared with the invaded scrambled siRNA-transfected untreated cells were shown as a percentage in a bar graph. All experiments were done three times. A simple *t*-test was used to assess differences in the number of invaded cells between any two experimental conditions. *, statistically significant compared with scrambled si-RNA-transfected cells treated with HRG; *P *< 0.05 was considered statistically significant. **(C) **SKBR3 cells were treated with 50 ng/mL HRG or 50 ng/mL HRG plus 10 μM C75 for 36 hours. The changes in cell invasion compared with the invaded HRG-induced cell invasion were shown as a percentage in a bar graph. All experiments were done three times. A simple *t*-test was used to assess differences in the number of invaded cells between any two experimental conditions. *, statistically significant compared with cells treated with HRG; *P *< 0.05 was considered statistically significant. Click here for file

Additional file 4 **Cell invasion in SKBR3 cells treated with C75 or lapatinib**. Cells were trypsinized and seeded at a density of 1 × 10^5 ^per insert. To investigate the role of lapatinib in cell invasion, we treated SKBR3 cells with untreated control or 0.2 μM lapatinib. To investigate the role of C75 in cell invasion, we treated both SKBR3 and BT474 cells with an untreated control or 10 μM C75. To evaluate the role of si-FASN in cell invasion, we transfected cells with scrambled siRNA or si-FASN for 24 hours. After 12 hours of serum starvation, the transfected cells were trypsinized and seeded at a density of 1 × 10^5 ^per insert and then incubated in serum-free medium. In the bottom well, 10% fetal bovine serum medium was used as a chemoattractant. After 36 hours of incubation, the invaded cells were stained with crystal violet and counted under a microscope. Representative microscopic images are shown. Invasion in treated cells is shown in a bar graph as a percentage of untreated (control) cells. All experiments were done three times. Click here for file

Additional file 5 **Cell invasion in BT474 cells treated with C75 or lapatinib**. Cells were trypsinized and seeded at a density of 1 × 10^5 ^per insert. To investigate the role of lapatinib in cell invasion, we treated cells with untreated control or 0.2 μM lapatinib. To investigate the role of C75 in cell invasion, we treated both SKBR3 and BT474 cells with an untreated control or 10 μM C75. To evaluate the role of si-FASN in cell invasion, we transfected cells with scrambled siRNA or si-FASN for 24 hours. After 12 hours of serum starvation, the transfected cells were trypsinized and seeded at a density of 1 × 10^5 ^per insert and then incubated in serum-free medium. In the bottom well, 10% fetal bovine serum medium was used as a chemoattractant. After 36 hours of incubation, the invaded cells were stained with crystal violet and counted under a microscope. Representative microscopic images are shown. Invasion in treated cells is shown in a bar graph as a percentage of untreated (control) cells. All experiments were done three times. Click here for file
