# Supplementary material for: Fatty acid synthase phosphorylation: a novel therapeutic target in HER2-overexpressing breast cancer cells
Source: Breast Cancer Res. 2010 Nov 16;12(6):R96. doi: 10.1186/bcr2777 (PMC3046439; doi:10.1186/bcr2777)
Supplement: Additional file 1 — Protein tyrosine phosphorylation in HER2-positive breast cancer cells treated with lapatinib. (A and B) SKBR3 breast cancer cells were treated with 1 μM lapatinib or DMSO (control) overnight (18 hours). Equal amounts of protein from total whole-cell lysates were immunoprecipitated with the antiphosphotyrosine antibody PT66 and separated by sodium dodecyl sulfate polyacrylamide gel electrophoresis. Steady-state protein levels of phosphotyrosine were assessed by silver staining (A) and Western blotting (B). (C) Phosphotyrosine immunoprecipitated complexes were obtained from SKBR3 and BT474 cells and detected by Western blotting on FASN. [file bcr2777-S1.PPT]

## Slide 1
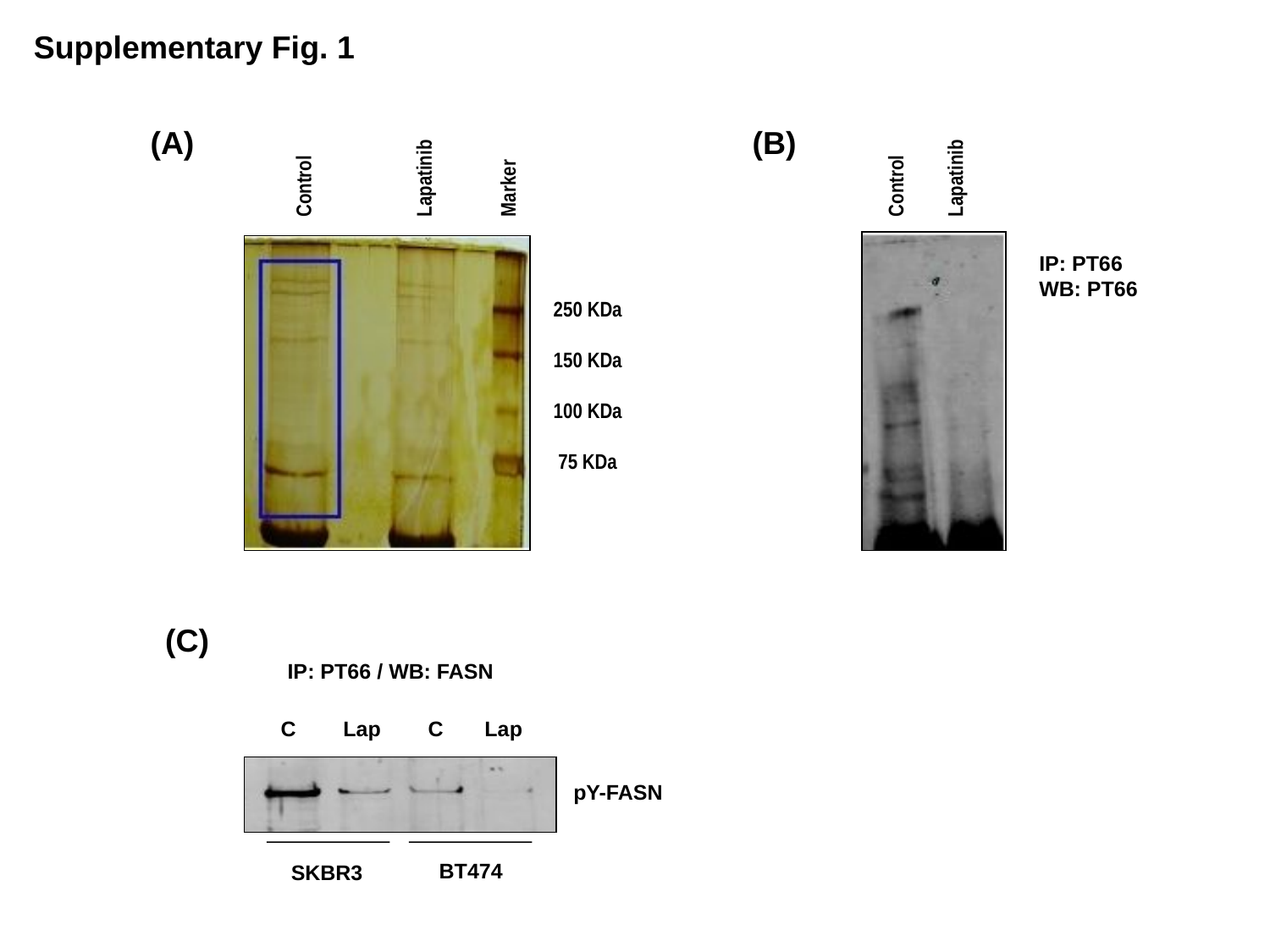

Supplementary Fig. 1
(A)
(B)
Control
Lapatinib
Control
Lapatinib
Marker
IP: PT66
WB: PT66
250 KDa
150 KDa
100 KDa
 75 KDa
(C)
IP: PT66 / WB: FASN
C Lap C Lap
pY-FASN
BT474
SKBR3
