# Supplementary material for: Fatty acid synthase phosphorylation: a novel therapeutic target in HER2-overexpressing breast cancer cells
Source: Breast Cancer Res. 2010 Nov 16;12(6):R96. doi: 10.1186/bcr2777 (PMC3046439; doi:10.1186/bcr2777)
Supplement: Additional file 2 — Viability of cells treated with low doses of lapabinib or C75. SKBR3 and BT474 cells were seeded at a density of 8 × 10^3^ cells/well on a 96-well plate. The next day, cells were treated with 0.2 μM lapatinib or 10 μM C75 with or without 50 ng/mL HRG for an additional 36 hours. Cell viability assay was performed by the use of the CellTiter-Glo luminescent cell viability assay kit (Promega Corporation) according to the manufacturer's instructions. [file bcr2777-S2.PPT]

## Slide 1
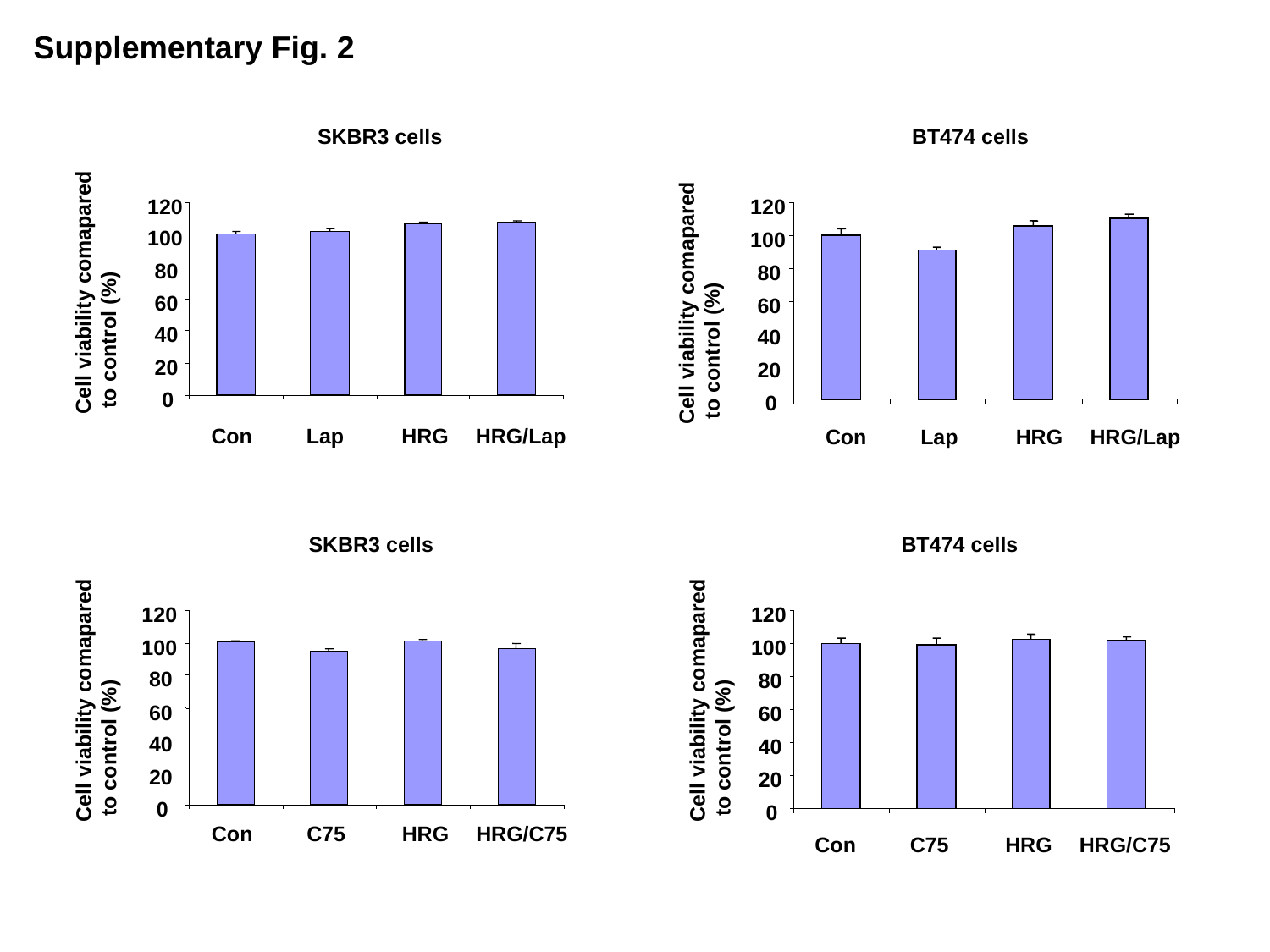

Supplementary Fig. 2
SKBR3 cells
BT474 cells
120
100
80
Cell viability comapared
 to control (%)
60
40
20
0
Con
Lap
HRG
HRG/Lap
120
100
80
60
40
20
0
Cell viability comapared
 to control (%)
Con
Lap
HRG
HRG/Lap
SKBR3 cells
BT474 cells
120
100
80
Cell viability comapared
 to control (%)
60
40
20
0
Con
C75
HRG
HRG/C75
120
100
80
60
40
20
0
Cell viability comapared
 to control (%)
Con
C75
HRG
HRG/C75
