# Supplementary material for: Fatty acid synthase phosphorylation: a novel therapeutic target in HER2-overexpressing breast cancer cells
Source: Breast Cancer Res. 2010 Nov 16;12(6):R96. doi: 10.1186/bcr2777 (PMC3046439; doi:10.1186/bcr2777)
Supplement: Additional file 3 — Implication of FASN and HER2 in the invasion of HER2-positive SKBR3 breast cancer cells. We assessed cell invasion using a matrigel invasion chamber as described in Materials and methods. (A) SKBR3 cells were treated with 50 ng/mL HRG or 50 ng/mL HRG plus 0.2 μM lapatinib for 36 hours. The changes in cell invasion compared with HRG-induced cell invasion were shown as a percentage in a bar graph. All experiments were done three times. A simple t-test was used to assess differences in the number of invaded cells between any two experimental conditions. *, statistically significant compared with cells treated with HRG; P < 0.05 was considered statistically significant. (B) SKBR3 cells transfected with either scrambled siRNA or si-FASN were treated with 50 ng/mL HRG for 36 hours. The efficiency of si-FASN knockdown was confirmed by Western blotting for FASN and β-actin. The changes in cell invasion compared with the invaded scrambled siRNA-transfected untreated cells were shown as a percentage in a bar graph. All experiments were done three times. A simple t-test was used to assess differences in the number of invaded cells between any two experimental conditions. *, statistically significant compared with scrambled si-RNA-transfected cells treated with HRG; P < 0.05 was considered statistically significant. (C) SKBR3 cells were treated with 50 ng/mL HRG or 50 ng/mL HRG plus 10 μM C75 for 36 hours. The changes in cell invasion compared with the invaded HRG-induced cell invasion were shown as a percentage in a bar graph. All experiments were done three times. A simple t-test was used to assess differences in the number of invaded cells between any two experimental conditions. *, statistically significant compared with cells treated with HRG; P < 0.05 was considered statistically significant. [file bcr2777-S3.PPT]

## Slide 1
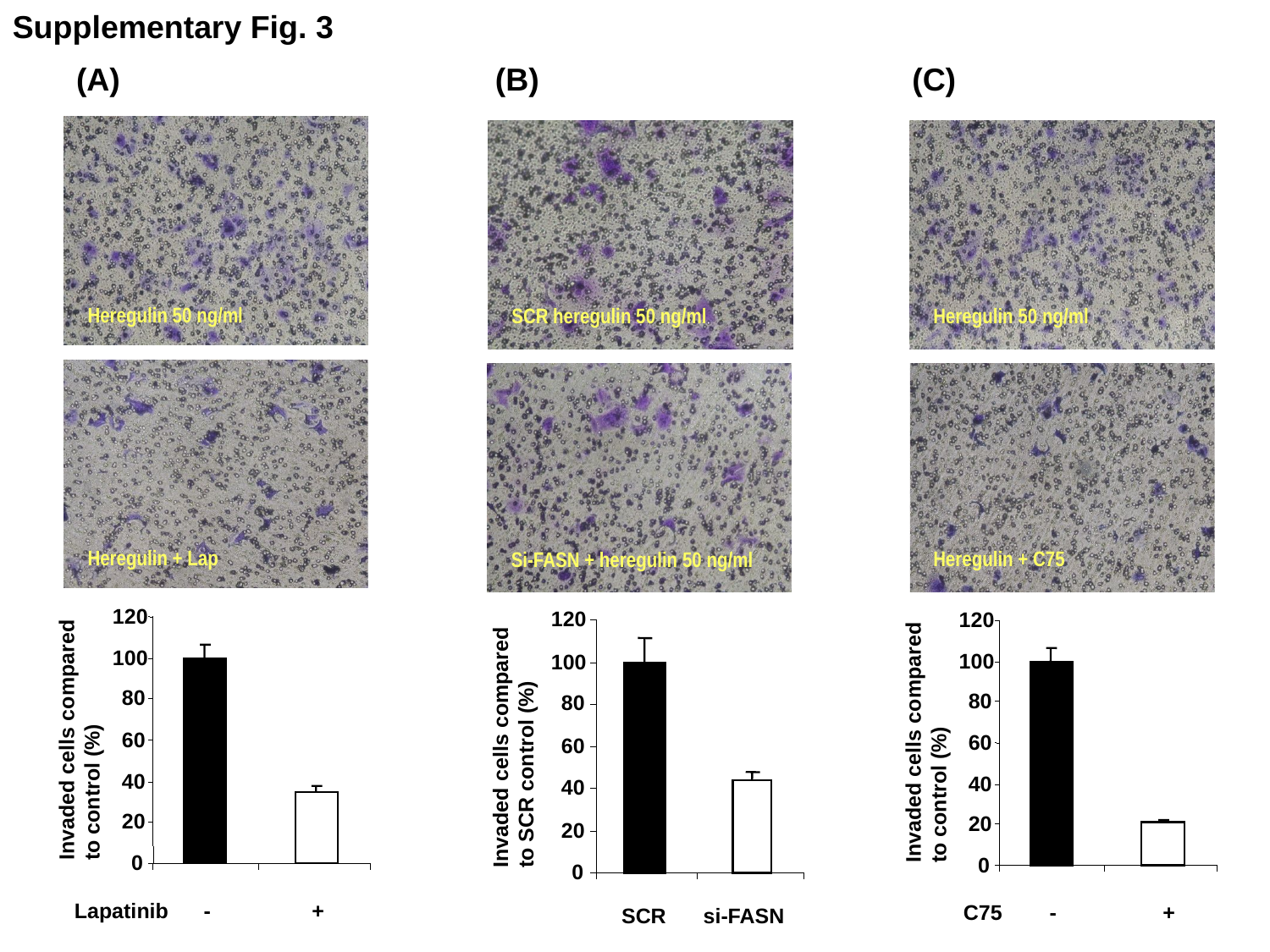

Supplementary Fig. 3
(A)
(B)
(C)
Heregulin 50 ng/ml
SCR heregulin 50 ng/ml
Heregulin 50 ng/ml
Heregulin + Lap
Si-FASN + heregulin 50 ng/ml
Heregulin + C75
120
100
80
Invaded cells compared
to control (%)
60
40
20
0
Lapatinib - +
120
100
80
Invaded cells compared
to SCR control (%)
60
40
20
0
SCR
si-FASN
120
100
80
Invaded cells compared
to control (%)
60
40
20
0
 C75 - +
