# Supplementary material for: Fatty acid synthase phosphorylation: a novel therapeutic target in HER2-overexpressing breast cancer cells
Source: Breast Cancer Res. 2010 Nov 16;12(6):R96. doi: 10.1186/bcr2777 (PMC3046439; doi:10.1186/bcr2777)
Supplement: Additional file 4 — Cell invasion in SKBR3 cells treated with C75 or lapatinib. Cells were trypsinized and seeded at a density of 1 × 10^5^ per insert. To investigate the role of lapatinib in cell invasion, we treated SKBR3 cells with untreated control or 0.2 μM lapatinib. To investigate the role of C75 in cell invasion, we treated both SKBR3 and BT474 cells with an untreated control or 10 μM C75. To evaluate the role of si-FASN in cell invasion, we transfected cells with scrambled siRNA or si-FASN for 24 hours. After 12 hours of serum starvation, the transfected cells were trypsinized and seeded at a density of 1 × 10^5^ per insert and then incubated in serum-free medium. In the bottom well, 10% fetal bovine serum medium was used as a chemoattractant. After 36 hours of incubation, the invaded cells were stained with crystal violet and counted under a microscope. Representative microscopic images are shown. Invasion in treated cells is shown in a bar graph as a percentage of untreated (control) cells. All experiments were done three times. [file bcr2777-S4.PPT]

## Slide 1
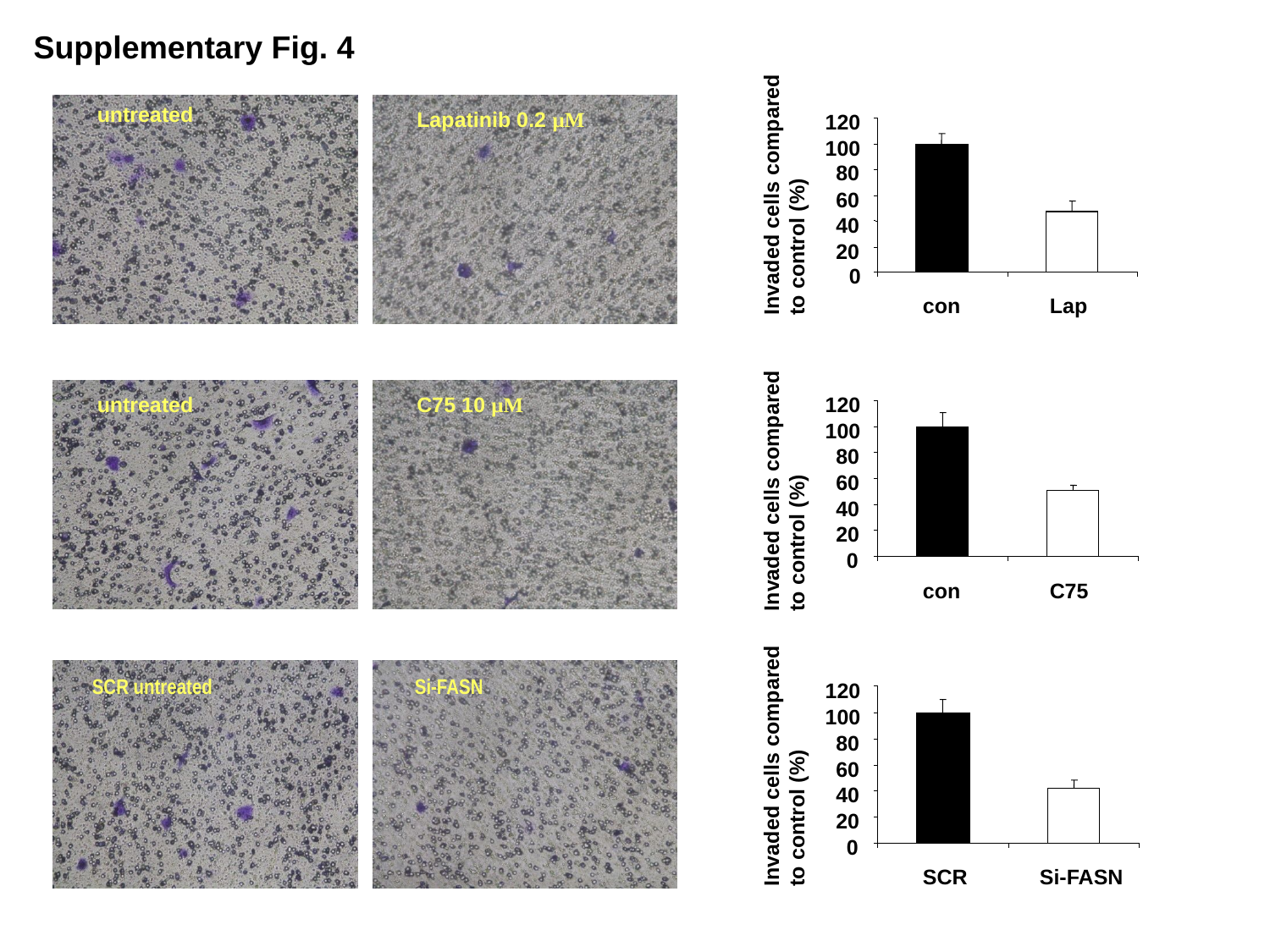

Supplementary Fig. 4
untreated
Lapatinib 0.2 μM
120
100
80
60
40
20
0
Invaded cells compared to control (%)
con
Lap
untreated
C75 10 μM
120
100
80
60
40
20
0
Invaded cells compared to control (%)
con
C75
SCR untreated
Si-FASN
120
100
80
60
40
20
0
Invaded cells compared to control (%)
SCR
Si-FASN
